# Comparative Analysis of Transcriptional Profiles of Adult *Schistosoma japonicum* from Different Laboratory Animals and the Natural Host, Water Buffalo

**DOI:** 10.1371/journal.pntd.0003993

**Published:** 2015-08-18

**Authors:** Shuai Liu, Xiaosu Zhou, Xianyu Piao, Chuang Wu, Nan Hou, Qijun Chen

**Affiliations:** 1 MOH Key Laboratory of Systems Biology of Pathogens, Institute of Pathogen Biology, Chinese Academy of Medical Sciences and Peking Union Medical College, Beijing, China; 2 Key Laboratory of Zoonosis, Jilin University, Changchun, China; Institute of Tropical Medicine (NEKKEN), JAPAN

## Abstract

**Background:**

Schistosomiasis is one of the most widely distributed parasitic diseases in the world. *Schistosoma japonicum*, a zoonotic parasite with a wide range of mammalian hosts, is one of the major pathogens of this disease. Although numerous studies on schistosomiasis japonica have been performed using laboratory animal models, systematic comparative analysis of whole-genome expression profiles in parasites from different laboratory animals and nature mammalian hosts is lacking to date.

**Methodology/Principal Findings:**

Adult schistosomes were obtained from laboratory animals BALB/c mice, C57BL/6 mice, New Zealand white rabbits and the natural host, water buffaloes. The gene expression profiles of schistosomes from these animals were obtained and compared by genome-wide oligonucleotide microarray analysis. The results revealed that the gene expression profiles of schistosomes from different laboratory animals and buffaloes were highly consistent (r>0.98) genome-wide. Meanwhile, a total of 450 genes were identified to be differentially expressed in schistosomes which can be clustered into six groups. Pathway analysis revealed that these genes were mainly involved in multiple signal transduction pathways, amino acid, energy, nucleotide and lipid metabolism. We also identified a group of 1,540 abundantly and stably expressed gene products in adult worms, including a panel of 179 *Schistosoma*- or Platyhelminthes-specific genes that may be essential for parasitism and may be regarded as novel potential anti-parasite intervention targets for future research.

**Conclusions/Significance:**

This study provides a comprehensive database of gene expression profiles of schistosomes derived from different laboratory animals and water buffaloes. An expanded number of genes potentially affecting the development of schistosomes in different animals were identified. These findings lay the foundation for schistosomiasis research in different laboratory animals and natural hosts at the transcriptional level and provide a valuable resource for screening anti-schistosomal intervention targets.

## Author Summary

The zoonotic parasite *Schistosoma japonicum* is one of the major pathogens of schistosomiasis and can parasitize a wide range of mammals. Although numerous schistosome transcriptional profiling studies have been performed using laboratory animal models, the differences in the global gene expression profiles of worms from different laboratory animals and natural mammalian hosts have not been characterized. Therefore, we studied the gene expression profiles of adult worms from BALB/c mice, C57BL/6 mice, rabbits and buffaloes using a transcriptomics approach. Our results indicate that, although the expression profiles of adult worms from different mammals are generally similar, hundreds of genes are differentially expressed, which were mainly involved in various signal transduction pathways, amino acid, energy, nucleotide and lipid metabolism. Numerous abundantly and stably expressed genes in adults were identified, including some genes that are only found in blood flukes or expanded within the phylum Platyhelminthes and may be important for parasitism. Our data provide a basis for schistosomiasis research in different mammalian hosts at the transcriptional level as well as a valuable resource for the screening of anti-schistosomal intervention targets.

## Introduction

Schistosomiasis is one of the most serious parasitic diseases and affects more than 200 million people globally, based on conservative estimates [1,2]. Schistosomiasis is caused mainly by three *Schistosoma* species: *Schistosoma japonicum*, *Schistosoma mansoni*, and *Schistosoma haematobium*. *S*. *japonicum* is endemic in Asia, principally China and the Philippines, whereas *S*. *mansoni* and *S*. *haematobium* are distributed in Africa and the Middle East. *S*. *mansoni* is also prevalent in South America [3]. Schistosomes have a complex developmental life cycle and exhibit sexual dimorphism. The life cycle comprises seven morphologically discrete stages (i.e., egg, miracidium, mother sporocyst, daughter sporocyst, cercaria, juvenile schistosomulum, and adult worm), complicating the control and prevention of schistosomiasis [4]. In contrast to the other two *Schistosoma* species known to infect humans, *S*. *japonicum* is a true zoonotic parasite that utilizes a wide range of mammalians as definitive hosts, including bovines, mice, rabbits, goats, pigs, and dogs [5,6].

Previous studies have indicated that the susceptibility of different mammalian hosts to *S*. *japonicum* infection varies; water buffaloes, rats, horses, and pigs are less susceptible to infection than mice, rabbits, yellow cattle, and goats [5]. Schistosomes derived from different mammalian hosts also exhibit visible changes in morphology, such as the length and width of worms, tegument, sucker and gynoecophorus [7,8]. In the last decade, a number of gene expression profiling studies of schistosomes have been conducted using various analytical approaches, and the findings have tremendously facilitated improved understanding of the molecular basis of schistosome developmental biology, host-parasite interactions and the pathogenesis of schistosomiasis (see more in reviews [9–11]). The majority of schistosomes used in those studies were isolated from laboratory animals such as BALB/c mice, C57BL/6 mice, and New Zealand white rabbits.

Recently, numerous differentially expressed genes that may influence parasite survival and development were identified by comparative proteomic analyses [12] and microarray analysis [13] of schistosomula from susceptible BALB/c mice, less susceptible Wistar rats and resistant reed voles. Yang et al. comparatively analyzed the gene expression profiles of *S*. *japonicum* derived from natural reservoir host yellow cattle, goats and water buffaloes using microarrays, and suggested that the gene expression patterns of some genes in schistosomes in natural hosts and laboratory animals may be diverse [7,8]. Data obtained using laboratory animals may not be completely transferable to natural hosts. A comparison of the gene expression profile of *S*. *japonicum* in its natural reservoir hosts and laboratory animals will undoubtedly facilitate our understanding of parasite biology. Previous epidemiological studies have revealed that bovines, particularly water buffaloes, are the major natural reservoir of *S*. *japonicum* and play a vital role in schistosomiasis transmission in China [14–17]. Therefore, we performed comparative analyses of the gene expression profiles of *S*. *japonicum* from the natural reservoir host, water buffalo, with those from laboratory animal mice and rabbits using a genome-wide microarray approach. Our results will be of significance for the screening of anti-schistosome targets and vaccine candidates using laboratory animals to further facilitate the control of schistosomiasis in natural reservoir hosts in endemic areas.

## Materials and Methods

### Ethical statement

All procedures performed on animals in this study were conducted following animal husbandry guidelines of the Chinese Academy of Medical Sciences and with permission from the Experimental Animal Committee with the Ethical Clearance Number IPB-2011-6.

### Parasite material

*S*. *japonicum*-infected *Oncomelania hupensis* were provided by the Hunan Institute of Parasitic Diseases, Yueyang, China. Laboratory BALB/c mice (nine mice), C57BL/6 mice (nine mice), New Zealand white rabbits (three rabbits) and natural reservoir host water buffaloes (three buffaloes) were infected with 40–400 freshly released cercariae through the upper back using the cover glass method [18]. Adult worms were perfused out the hepatic portal vein of infected animals at approximately 7 weeks, washed briefly with PBS and soaked immediately in RNAlater Solution (Ambion, CA, USA) and stored at -80°C until total RNA was extracted.

### Total RNA isolation

Total RNA was extracted from worms from different animals using the RNeasy Mini kit (QIAGEN), and contaminating genomic DNA was removed from RNA samples using a DNA-free kit (Ambion, CA, USA). The quantity and quality of the RNA samples were assessed using a ND-1000 spectrophotometer (NanoDrop Technologies, Wilmington, DE) and denaturing agarose gel electrophoresis.

### Microarray construction, hybridization and data analysis

Schistosome genome-wide microarrays were used to analyze the gene expression profiles of *S*. *japonicum* derived from different laboratory animals and the natural host, water buffalo. The design and construction of the microarray and the methods used in microarray hybridization and feature extraction have been previously reported [19–21]. The full details of this schistosome microarray design have been deposited in the Gene Expression Omnibus public database (http://www.ncbi.nlm.nih.gov/geo) under platform accession number GPL18617. Briefly, a total of 21,861 target sequences (20,194 sequences derived from *S*. *japonicum* and 1667 sequences derived from *S*. *mansoni*) were provided to Roche NimbleGen for array design. For each sequence, 3 or 4 60-mer oligonucleotide probes were designed. Probes with random sequences were printed as negative control (background signal) and eight spike-RNA probes from the intergenic sequence of yeast as hybridization controls. The microarray was manufactured by Roche NimbleGen. Microarrays were printed in a 12×135 K feature format. cDNA was labeled with a fluorescent dye (Cy3-dCTP) using cRNA Amplification and Labeling Kit (CapitalBio, Beijing, China). Hybridization was performed using three biological replicates for all samples at CapitalBio. Procedures of array hybridization, washing, scanning, and data acquisition were carried out according to the NimbleGen Arrays user’s guide. The arrays were scanned using MS200 scanner (NimbleGen Systems) with 2 μm resolution, and NimbleScan software (NimbleGen) was used to extract fluorescent intensity raw data from the scanned images. The normalized expression data of genes was generated using the Robust Multichip Average (RMA) algorithm [22–24], which consisted of three steps: a background adjustment, quantile normalization and final summarization. The outlier probes were identified and the contribution of outlier probes was reduced in the reported gene expression level, which has been demonstrated to improve the sensitivity and reproducibility of microarray results. Then, the expression value of a gene is a weighted average of all probes when both background correction and quantile normalization were performed. Raw data and normalized gene level data from the arrays have been deposited at the public database Gene Expression Omnibus under the accession number GSE65327. Genes were considered differentially expressed by expression fold-change (FC) ≥2 between any two compared worm samples and *p*<0.05 (Student’s *t*-test). The stably and abundantly expressed genes among the adult worms from the four mammalian hosts were extracted using coefficient of variation (CV) ≤0.1 and fluorescence intensity ≥10,000 on the basis of retrieval of a panel of well characterized *Schistosoma* genes of which the intensity values were greater than 10,000. Hierarchical clustering analysis of selected genes was performed to generate heat maps using the software Cluster 3.0 [25] and Heatmap Builder 1.0 [26].

### Real-time PCR

A subset of genes with different expression patterns were selected for further validation using real-time PCR as previously described [19]. Real-time PCR primers were designed using Primer Express 3.0 software (Applied Biosystems, Foster City, USA) (S1 Table). For each sample, 1 μg of total RNA was used to synthesize the first strand cDNA using a Reverse Transcriptase Kit (Invitrogen, CA, USA) with oligo (dT) 12–18 primers (Invitrogen) in a final volume of 20 μl. PCR reactions were performed in technological triplicate on a 7300 Real-Time PCR system (Applied Biosystems) using SYBR Green QPCR Master Mix (Agilent Technologies, USA) according to the manufacturer’s instructions. The 26S proteasome non-ATPase regulatory subunit 4 gene (PSMD4, GenBank Accession No. FN320595), which has been validated as a reliable reference gene in transcriptomic analysis of *S*. *japonicum*, was employed as a reference gene in the real-time PCR analysis [19]. The relative expression level of each gene was analyzed using SDS 1.4 software (Applied Biosystems).

### Gene ontology and pathway analyses

Gene sequences were functionally annotated using Blast2GO [27], and the output provided combined graphics for three categories of gene ontology (GO) terms: biological processes, molecular functions, and cellular components. The Kyoto Encyclopedia of Genes and Genomes (KEGG) automated annotation server was used to assign pathway-based functional orthology to differentially expressed genes [28]. Signal peptides were predicted using the program SignalP 4.1 server employing both the neural network and hidden Markov model [29], and transmembrane helices were predicted using TMHMM 2.0 [30].

### Identification and phylogenetic analysis of *S*. *japonicum* venom-allergen-like protein (SjVAL) genes

SjVAL genes were identified using the BLASTp and InterProScan [31] algorithms. Initially, the sequences of *S*. *mansoni* venom-allergen-like proteins (SmVALs) [32] were used for BLASTp searches of *S*. *japonicum* predicted protein sequence database [33] and the non-redundant protein sequence database [34,35] of the National Center for Biotechnology Information (NCBI) (e-value cut-off: 10^−5^). CD-HIT v4.5.4 software (http://www.bioinformatics.org/cd-hit/) was then used to eliminate redundant protein sequences from the obtained putative SjVALs using the criteria of 95% identity and 80% coverage between two sequences. Finally, all remaining protein sequences were confirmed by the presence of SCP/TAPS-representative protein domains (IPR014044) using InterProScan [31]. A phylogenetic relationship tree was built using the full-length amino acid sequences of *Schistosoma* VALs in the following steps. First, sequences were aligned using ClustalX [36] and then refined manually, and a phylogenetic tree was finally generated using MEGA 5.0 software [37] by the neighbor-joining (NJ) method (the bootstrap test was performed with 1000 replicates).

### Accession numbers

Accession numbers for the *S*. *japonicum* sequences used in the alignment are: CAX74321.1, CAX74107.1, CAX73316.1, CAX78430.1, CAX72962.1, CAX76177.1, CAX73488.1, CAX78439.1, CAX78429.1, CAX78435.1, AAP06001.1, AAW25717.1, AAW25499.1, AAW25247.1, AAW25007.1, AAW27353.1, Sjp_0038950, Sjp_0038960, Sjp_0083700 and Sjp_0112690. Accession numbers for the *S*. *mansoni* sequences used in the alignment are: AAY43180.1 (SmVAL1), AAY43181.1 (SmVAL2), AAZ04923.2 (SmVAL3), AAY43182.1 (SmVAL4), ABB88846.2 (SmVAL5), CCD74794.1 (SmVAL6), AAZ04924.1 (SmVAL7), ABW98681.1 (SmVAL8), ABB88845.1 (SmVAL9), ABO09814.2 (SmVAL10), ABA54555.1 (SmVAL11), ABB88844.1 (SmVAL12), ABB88843.1 (SmVAL13), ABO09815.1 (SmVAL14), CCD80670.1 (SmVAL15), CCD74792.1 (SmVAL16), CCD74934.1 (SmVAL17), CCD80318.1 (SmVAL18), CCD80317.1 (SmVAL19), CCD80812.1 (SmVAL20), CCD80564.1 (SmVAL21), CCD59744.1 (SmVAL22), CCD80666.1 (SmVAL24), CCD80667.1 (SmVAL25), CCD80638.1 (SmVAL26), CCD80648.1 (SmVAL27) and CCD80636.1 (SmVAL28).

## Results and Discussion

### Correlation analysis of the transcriptional profiles of schistosomes from different laboratory animals and buffaloes

This study compared the transcriptional profiles of adult worms isolated from experimental animals (BALB/c mice, C57BL/6 mice and New Zealand white rabbits) and the natural host (water buffaloes) using genome-wide microarray analyses and combinatorial bioinformatics. First, we assessed a common correlation of all the genes within arrays between biological replicates of schistosomes from the same host to assess the quality of the biological replicates. As expected, the correlation coefficient (r) between biological replicates was >0.99, demonstrating a strong consistency between the biological replicates of worms from the same host (Table 1). Similar results were obtained by correlation analyses of biological replicates for the global expression profiles of schistosomes from water buffaloes, yellow cattle and goats [7,8]. We then investigated the transcriptional profile correlation between worm samples from different hosts using an average of three biological replicates. The results demonstrated that the transcriptional profiles between worms from different hosts were also highly similar as a whole (r>0.98, Table 2).

**Table 1:** Correlation analysis of gene expression profiles between the biological triplicates.

Biological replicates	C57BL/6	BALB/c	Rabbit	Buffalo
1 and 2	0.992	0.995	0.996	0.998
1 and 3	0.997	0.998	0.998	0.998
2 and 3	0.995	0.996	0.998	0.998

**Table 2:** Correlation analysis of gene expression profiles between schistosomes from BALB/c mice, C57BL/6 mice, rabbits and buffaloes.

Different hosts	C57BL/6	BALB/c	Rabbit	Buffalo
C57BL/6	1	0.998	0.989	0.992
BALB/c	0.998	1	0.991	0.994
Rabbit	0.989	0.991	1	0.995
Buffalo	0.992	0.994	0.995	1

### Pairwise comparisons of the gene expression profiles of schistosomes from different laboratory animals and buffalo

**Fig 1:**
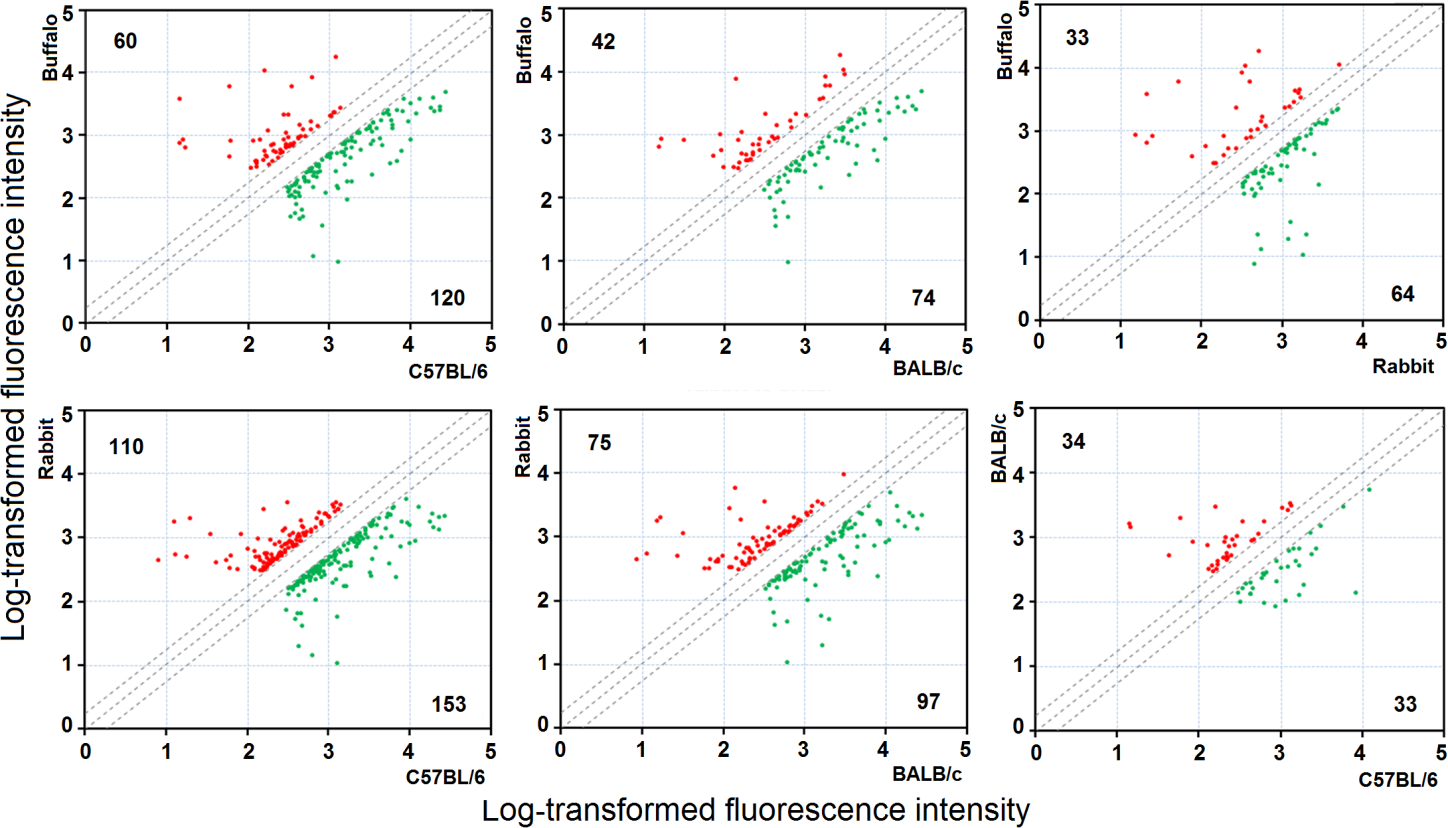
Scatter plots of pairwise comparisons between schistosomes from BALB/c mice, C57BL/6 mice, rabbits and water buffaloes. The *x*-axis and *y*-axis correspond to the log-transformed fluorescent intensity values of genes in the pairwise worm samples. The red plots and green plots represent up-regulated genes and down-regulated genes, respectively (FC≥2, *p*<0.05). The number of up-regulated genes or down-regulated genes is shown at the upper left and lower right, respectively.

A series of pairwise comparisons between schistosomes isolated from the four mammalian hosts was performed to identify differentially expressed genes. Using FC≥2 and *p*<0.05, subsets of differentially expressed gene products were identified: 180 (buffalo vs. C57BL/6), 116 (buffalo vs. BALB/c), 97 (buffalo vs. rabbit), 263 (rabbit vs. C57BL/6), 172 (rabbit vs. BALB/c) and 67 (C57BL/6 vs. BALB/c). The distributions of the up- and down-regulated genes between the paired comparisons are displayed as scatter plots (Fig 1 and S2 Table). It is understandable that adult worms from C57BL/6 and BALB/c mice shared more similar transcriptional profiles than those from rabbits and buffaloes due to the evolutionary distance between the two animal species. Previous studies have shown that fewer differentially expressed genes were identified in schistosomes between water buffalo and yellow cattle (69 genes) than between water buffalo and goat (485 genes) [7,8].

**Fig 2:**
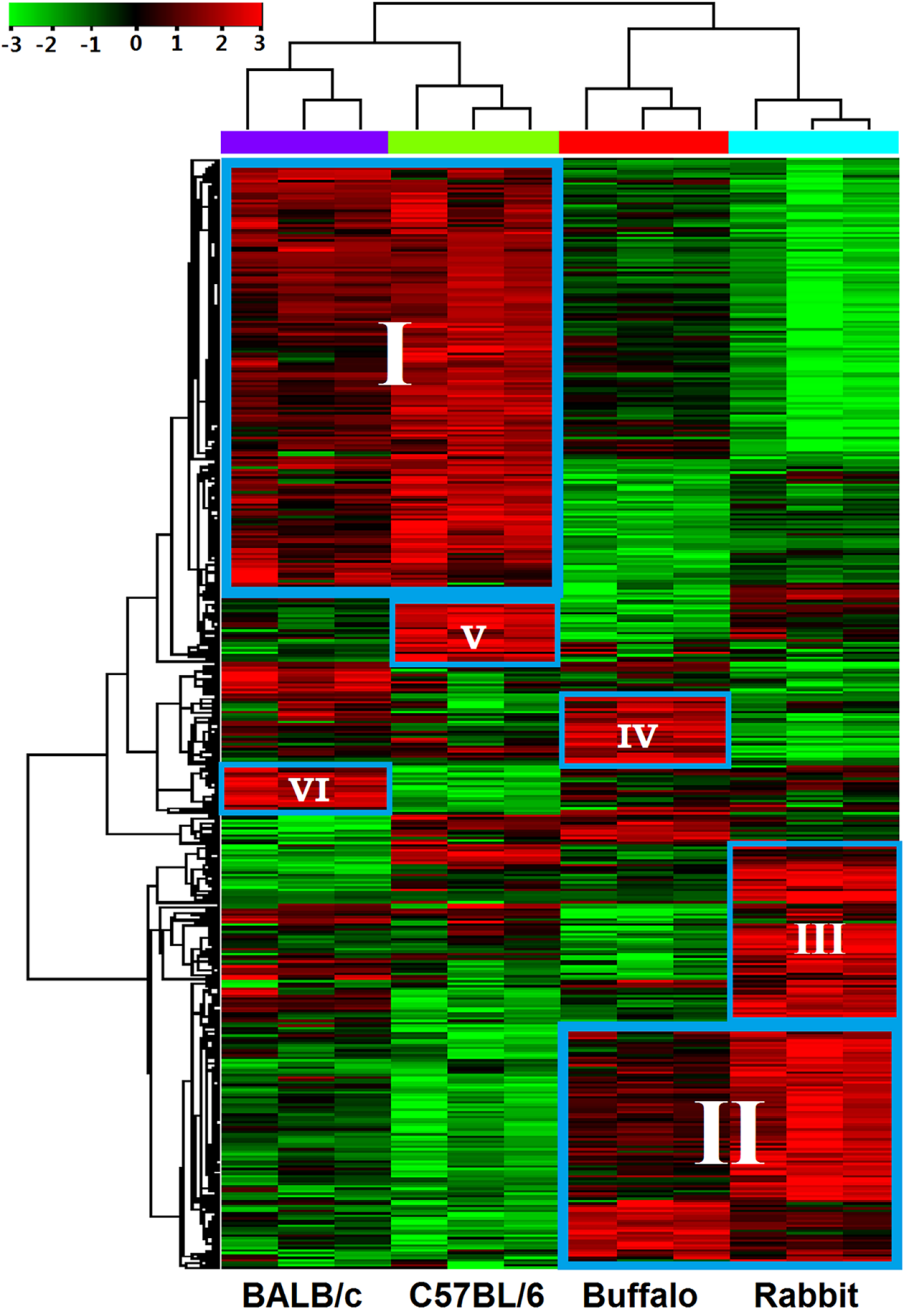
Hierarchical clustering of differentially expressed genes in worms from BALB/c mice, C57BL/6 mice, rabbits and water buffaloes. The heat map of 450 differentially expressed genes extracted from the microarray dataset is shown (three biological replicates were performed for each worm sample). Six major clusters of differentially expressed genes were identified: cluster I, genes significantly expressed in worms from BALB/c mice and C57BL/6 mice; cluster II, genes highly expressed in worms from water buffaloes and rabbits; cluster III-VI, genes respectively up-regulated in the worms from rabbits, water buffaloes, C57BL/6 mice and BALB/c mice. The color scale represents relative expression levels: red, up-regulated; green, down-regulated; and black, unchanged.

**Fig 3:**
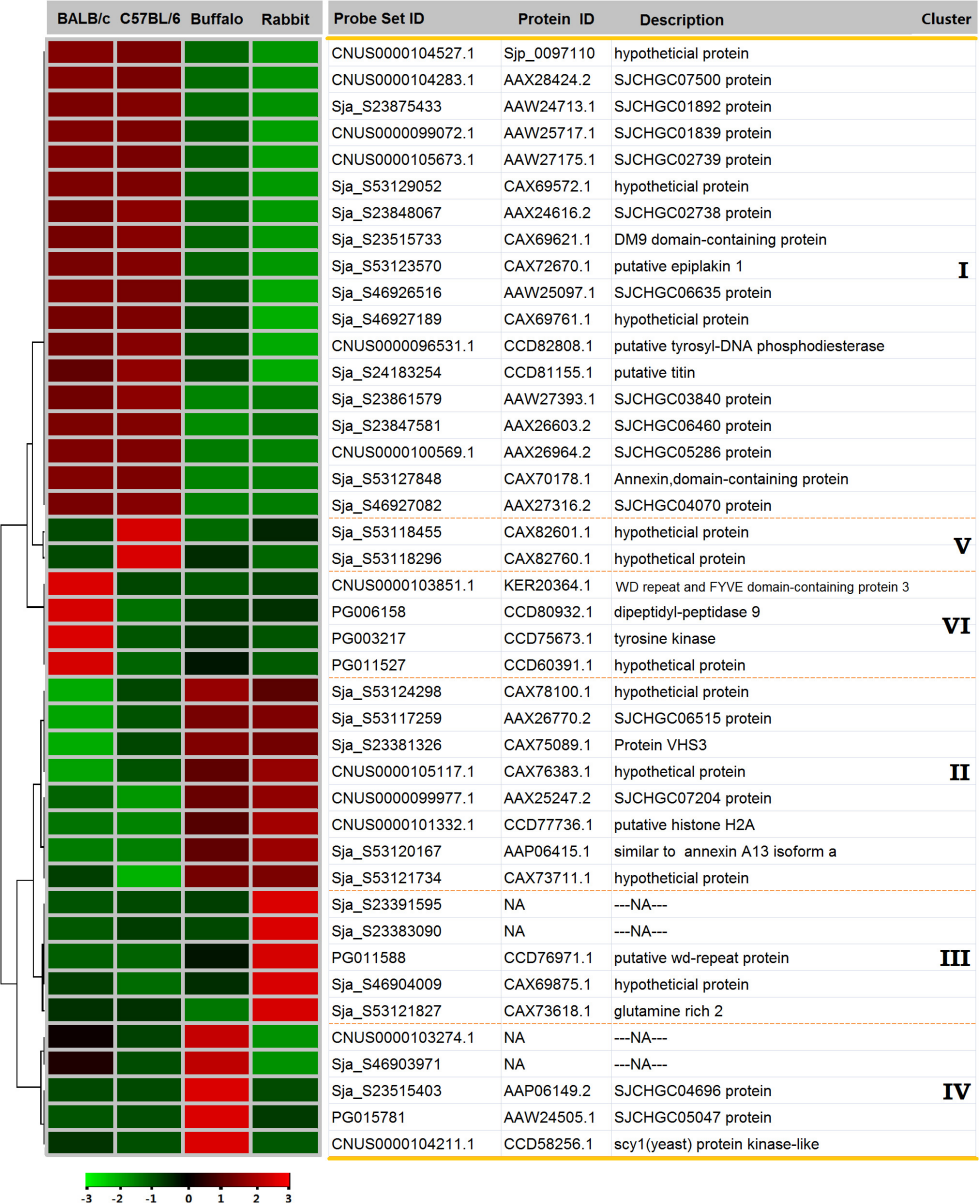
Examples of differentially expressed genes from the six clusters. The heat map illustrates the hierarchical clustering of 42 selected common differentially expressed genes using the average gene expression values of the three biological replicates. The color scale represents relative expression levels: red, up-regulated; green, down-regulated; and black, unchanged.

After integrating these subsets of differentially expressed genes, a total of 450 non-redundant genes were used for hierarchical clustering to enable collective visualization by gene and array (See supplementary S3 Table for details). As expected, these genes were clustered into four subgroups, of which genes of the biological replicates were clustered together, and branched by the host origination of the samples (Fig 2). The result further demonstrated high consistency between the biological replicates. The heat map demonstrated that these differentially expressed genes clustered into two major transcriptional patterns: cluster I, genes that were up-regulated in schistosomes isolated from C57BL/6 mice and BALB/c mice and down-regulated in schistosomes isolated from buffaloes and rabbits; and cluster II, genes that were down-regulated in schistosomes isolated from C57BL/6 mice and BALB/c mice and up-regulated in schistosomes isolated from buffaloes and rabbits (Fig 2). Meanwhile, several other clusters of differentially expressed genes were also identified by hierarchical clustering: cluster III, genes highly expressed in the worms from rabbits; cluster IV, genes up-regulated in worms from buffaloes; cluster V, genes up-regulated in worms from C57BL/6 mice; and cluster VI, genes highly expressed in worms from BALB/c mice (Fig 2). A list of representative differentially expressed genes from each cluster is displayed in detail as a heat map (Fig 3).

We also compared our results with previous studies on gene expression profiles of *S*. *japonicum* derived from the natural mammalian hosts, buffaloes, cattle and goats [7,8]. Some of these differentially expressed genes in adult schistosomes from the different hosts have been identified in previous studies, such as CNUS0000098059, CNUS0000105021, CNUS0000096235, CNUS0000102644, FN318955 and FN313838. Meanwhile, a number of differentially expressed genes identified by previous studies were not identified in our study since the fluorescence intensity values of these genes were below the cut-off value (S4 Table).

### Confirmation of microarray data by real-time PCR

**Fig 4:**
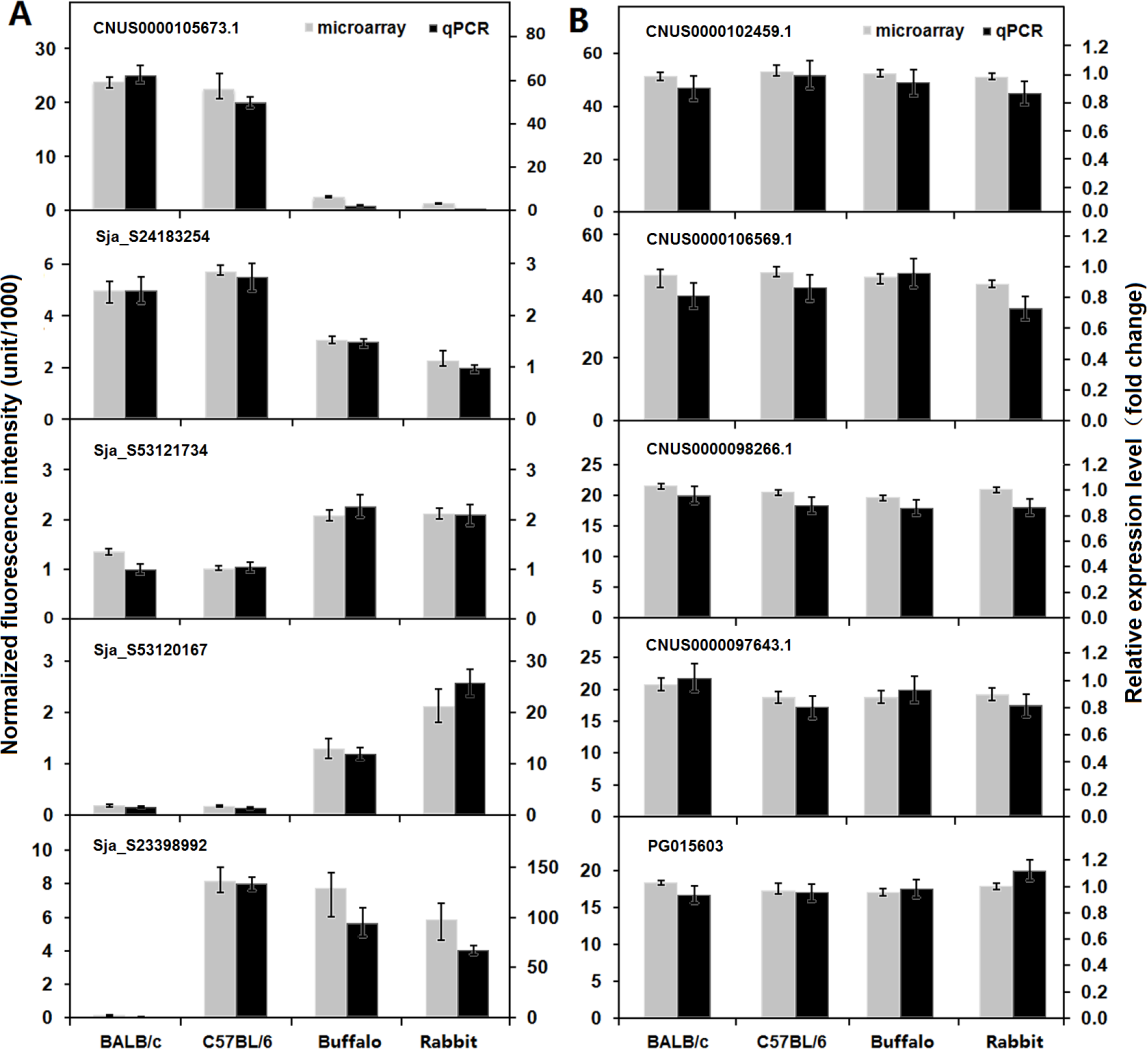
Validation of a subset of genes exhibiting different expression patterns. The expression patterns of ten selected genes in adult worms from BALB/c mice, C57BL/6 mice, rabbits and water buffaloes were quantified by real-time PCR analysis. **(A)** Genes differentially expressed in worms from the four mammalian hosts. **(B)** Genes stably expressed in worms from the four mammalian hosts. The corresponding microarray gene expression data are presented as the average gene expression values of three biological replicates, and the upper and lower error bars represent the maximum and minimum values of the three biological replicates. The relative expression levels of genes were calculated using SDS v1.4 software (Applied Biosystems) and the error bars represent the standard deviation for three technical replicates.

To validate the microarray transcription data, a subset of ten genes with various biological functions and different expression patterns was selected for real-time PCR validation. The real-time PCR results well matched the microarray data (Fig 4) with a significant correlation factor of 0.943 (Spearman’s Rho, *p*<0.0001, n = 40), thereby validating the microarray results.

### GO and KEGG pathway analyses of differentially expressed genes

**Fig 5:**
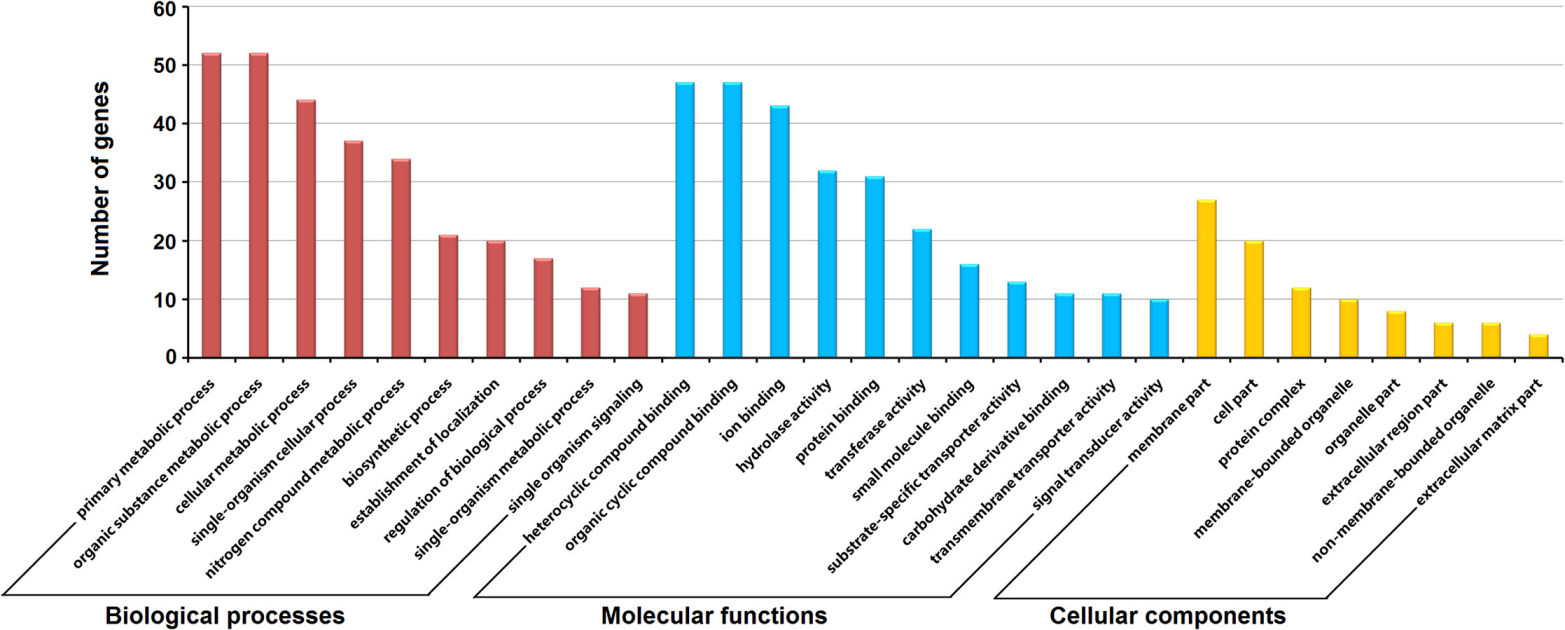
Gene ontology distributions of the differentially expressed genes in adult worms from BALB/c mice, C57BL/6 mice, rabbits and buffaloes. The Blast2Go program defined the GO terms in three categories: biological processes, molecular functions, and cellular components. Data without assigned GO terms were excluded from the graph.

GO analysis was performed to summarize and explore the major GO categories of the differentially expressed genes which may be susceptible to host environments. A total of 174 gene sequences were annotated with GO terms in three independent categories: biological processes (161 gene sequences), molecular functions (170 gene sequences), and cellular components (50 gene sequences) (Fig 5 and S3 Table). The biological processes analysis revealed that the predominant genes were involved in response to metabolic processes, including primary metabolic process, organic substance metabolic process, cellular metabolic process and nitrogen compound metabolic process. For molecular functions, the majority of genes were annotated with binding activity such as organic cyclic compound binding activities, heterocyclic compound binding activity, and ion binding activity. Notably, genes coding for proteins associated with membrane accounted for the major portion of annotated genes in the cellular components analysis.

Comparative genomics analysis of well characterized signaling pathways between schistosome and mammalian host indicates that schistosome genome encodes various growth factors, receptors and other critical components to regulate numerous cellular processes during tissue development and organogenesis. These components share high sequence similarity with mammalian orthologues, implying that schistosomes may utilize host components as developmental signals besides their own pathways [33]. The KEGG pathway analysis showed that many of these differentially expressed genes were involved in signaling transduction pathways, such as calcium signaling pathway, cAMP signaling pathway, PI3K-Akt signaling pathway, Rap1 signaling pathway, Ras signaling pathway, ErbB signaling pathway and MAPK signaling pathway (Table 3). Previous studies has also found that genes involved in ErbB signaling pathway and MAPK signaling pathway were differentially expressed in worms from buffaloes and goats [8]. Moreover, the *S*. *japonicum* genome reveals that schistosomes are not able to *de novo* synthesize fatty acids, sterols, purines, essential amino acids, which must be acquired from their hosts. Analysis of the KEGG pathways assigned to metabolic process indicated that these differentially expressed genes mainly participated in amino acid, energy, nucleotide and lipid metabolism (Table 3), which is consistent with the findings of Yang et al. [7]. All the results above further prove that schistosomes can exploit host nutrients and signaling pathways for growth and development, and host environments can affect the survival and development of the parasites. For instance, human tissue factor pathway inhibitor (TFPI), which acts as a plasma Kunitz-type serine protease inhibitor, is an anti-coagulation protein that plays an important role in the regulation of the blood coagulation cascade [38]. Interestingly, the *S*. *japonicum* TFPI gene (CAX69506.1) was significantly up-regulated in the intra-mammalian stage of the parasite life cycle and variously expressed in schistosomes from different hosts (S1 Fig). It will be engrossing to investigate the function of the TFPI gene in schistosomes in connection with the blood parasitic environment. Notably, one trematode eggshell synthesis protein gene (TES, pfam08034), AAW25913.1, was significantly up-regulated in worms from C57BL/6 mice, buffaloes and rabbits comparing with those from BALB/c mice. The trematode eggshell synthesis protein genes have been identified in several trematode parasites, which are crucial for eggshell synthesis, a key step for determining the quality and quantity of eggs laid [39]. Annexins are a multigene family of calcium-dependent phospholipid-binding proteins, and members of this family have been identified in major eukaryotic phyla [40]. In humans, annexins interact with various cell-membrane components by forming networks on the membrane surface that are involved in the regulation of membrane organization, cell differentiation and migration, intracellular signaling by enzyme modulation and ion fluxes [40–42]. Although annexins lack signal peptides for secretion, some extracellular members have been identified that act as receptors for serum proteases on the endothelium as well as inhibitors of neutrophil migration and blood coagulation [40]. In addition, some human annexin isoforms are involved in immunoregulatory functions such as the resolution of inflammation [43]. In *S*. *mansoni*, three annexin genes (Smp_074140, Smp_074150 and Smp_077720) have been identified by proteomic analysis as the membrane-associated constituents of the tegument surface [44]. Of the annexins, Smp_077720 is significantly up-regulated in the transition from free-living cercaria to parasitic schistosomulum and adult worm and binds to the tegument surface membranes in a calcium-dependent manner [45]. We identified three annexin domain-containing protein genes (AAX26603.2, CAX70178.1 and AAP06415.1) that were differentially expressed in schistosomes from the four different mammalian hosts. AAX26603.2 and CAX70178.1 were significantly up-regulated in worms from BALB/c and C57BL/6 mice compared with worms from buffaloes and rabbits, but the opposite expression pattern was observed for AAP06415.1 (Fig 3). This result indicates that these genes are either under different regulatory mechanisms or that the encoded proteins are functionally different. In addition, a gene coding for a putative endoribonuclease (AAX27316.2) containing the conserved endoribonuclease XendoU domain was significantly up-regulated in schistosomes from mice compared with those from buffaloes and rabbits. XendoU, which was first identified in *Xenopus laevis*, is a U-specific, metal ion-dependent endoribonuclease and is involved in the processing of intron-encoded small nucleolar RNAs (snoRNA) [46–48]. Schwarz et al. recently determined that the calcium-dependent endoribonuclease XendoU promotes endoplasmic reticulum network formation through local RNA degradation [49].

**Table 3:** Pathway mapping for selected differentially expressed genes in worms from BALB/c mice, C57BL/6 mice, rabbits and buffaloes.

Pathway	KEGG	Probe ID	Gene description	Fold change			
				Bc	C57	Bu	Ra
**Signal transduction**							
Calcium signaling pathway	K04962	CNUS0000095538.1	ryanodine receptor 2	1.8	2.0	1.4	1.0
	K05853	CNUS0000103090.1	Ca2+ transporting ATPase	2.0	2.1	1.6	1.0
	K04163	CNUS0000103128.1	5-hydroxytryptamine receptor 7	1.6	2.4	1.3	1.0
	K04634	CNUS0000095654.1	guanine nucleotide-binding protein G(q) subunit alpha	1.0	1.3	2.4	2.0
	K02183	PG003603	calmodulin	1.1	1.0	1.3	2.2
cAMP signaling pathway	K04962	CNUS0000095538.1	ryanodine receptor 2	1.8	2.0	1.4	1.0
	K04630	CNUS0000104373.1	guanine nucleotide-binding protein G(i) subunit alpha	1.2	2.3	1.0	1.5
	K02183	PG003603	calmodulin	1.1	1.0	1.3	2.2
PI3K-Akt signaling pathway	K06237	CNUS0000098613.1	collagen, type IV, alpha	2.6	3.0	1.5	1.0
	K06236	CNUS0000095675.1	collagen alpha-1(V)	1.8	2.0	1.6	1.0
	K06236	PG001771	collagen alpha-1(V)	1.7	2.0	1.4	1.0
Rap1 signaling pathway	K04634	CNUS0000095654.1	guanine nucleotide-binding protein G(q) subunit alpha	1.0	1.3	2.4	2.0
	K04630	CNUS0000104373.1	guanine nucleotide-binding protein G(i) subunit alpha	1.2	2.3	1.0	1.5
	K02183	PG003603	calmodulin	1.1	1.0	1.3	2.2
Ras signaling pathway	K06619	PG007218	abelson tyrosine-protein kinase 1	2.0	1.5	1.0	1.1
	K04163	CNUS0000103128.1	5-hydroxytryptamine receptor 7	1.6	2.4	1.3	1.0
	K02183	PG003603	calmodulin	1.1	1.0	1.3	2.2
ErbB signaling pathway	K06619	PG007218	abelson tyrosine-protein kinase 1	2.0	1.5	1.0	1.1
MAPK signaling pathway	K04437	CNUS0000095805.1	filamin	2.0	2.2	1.6	1.0
Hippo signaling pathway	K16175	CNUS0000096762.1	protein scribble	1.3	1.0	7.4	1.6
**Metabolism**							
Amino acid metabolism	K01593	CNUS0000102482.1	aromatic-L-amino-acid decarboxylase	3.0	4.5	1.0	2.9
	K00272	CNUS0000096157.1	D-aspartate oxidase	2.8	2.2	1.0	1.1
	K00472	Sja_S53123004	prolyl 4-hydroxylase	1.6	2.0	1.6	1.0
Energy metabolism	K02261	Sja_S23866441	cytochrome c oxidase subunit 2	64.8	133.4	1.0	1.1
	K00412	Sja_S23383389	ubiquinol-cytochrome c reductase cytochrome b subunit	1.4	1.2	1.0	163.4
	K02153	CNUS0000096235.1	V-type H+-transporting ATPase subunit e	1.7	2.4	1.5	1.0
Nucleotide metabolism	K16860	CNUS0000097921.1	DNA polymerase delta subunit 2	1.6	3.0	1.0	2.2
	K00760	Sja_S23873566	hypoxanthine phosphoribosyltransferase	2.7	3.5	3.1	1.0
Lipid metabolism	K16860	Sja_S23367919	phospholipase D3/4	1.8	2.2	1.8	1.0
Porphyrin and chlorophyll metabolism	K01719	PG011197	uroporphyrinogen-III synthase	1.6	1.0	1.8	2.9
Inositol phosphate metabolism	K10572	CNUS0000107328.1	inositol-pentakisphosphate 2-kinase	1.8	2.2	1.3	1.0

Microarray data are presented as fold changes relative to sample with the lowest intensity values. Bc, C57, Bu and Ra separately represent worms from BALB/c mice, C57BL/6 mice, buffaloes and rabbits.

**Fig 6:**
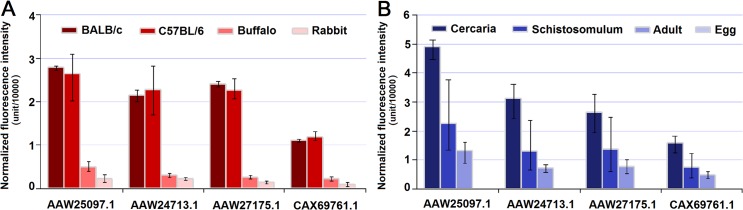
Transcription profiles of four *Schistosoma*-specific genes in worms from different mammalian hosts and developmental stages. **(A)** Transcription profiles of the four *Schistosoma*-specific genes in adult worms from BALB/c mice, C57BL/6 mice, water buffaloes and rabbits. **(B)** Transcription profiles of the four *Schistosoma*-specific genes in egg, schistosomulum, adult worm (from rabbits) and cercaria. The average gene expression values of three biological replicates were obtained from the microarray data. The upper and lower error bars represent the maximum and minimum values of the three biological replicates.

Notably, we observed that many of these differentially expressed genes were annotated as hypothetical proteins. Sequence alignment analysis revealed that these genes shared no sequence similarity to any sequence present in the non-redundant protein sequence (nr) databases of NCBI at a cut-off E value of 10^−5^ (except for *Schistosoma* species). Thus these *Schistosoma*-specific genes are likely specialised for parasitism by schistosomes, although their functions are unknown. Here, we identified a panel of four *Schistosoma*-specific genes (AAW25097.1, AAW27175.1, AAW24713.1 and CAX69761.1) that encoded a putative signal peptide and were overexpressed in worms from mice compared with those from buffaloes and rabbits. These four genes also exhibited a similar expression pattern among different developmental stages: significant down-regulation in the transition from free-living cercaria to parasitic schistosomulum and adult worm, and no expression in the egg stage (worms isolated from rabbits) (Fig 6). The intriguing expression patterns of these *Schistosoma*-specific genes spark interests in further characterization of their function and their potential contribution to successful parasitism.

### Stably and abundantly expressed genes in adult schistosomes from different laboratory animals and buffaloes

**Fig 7:**
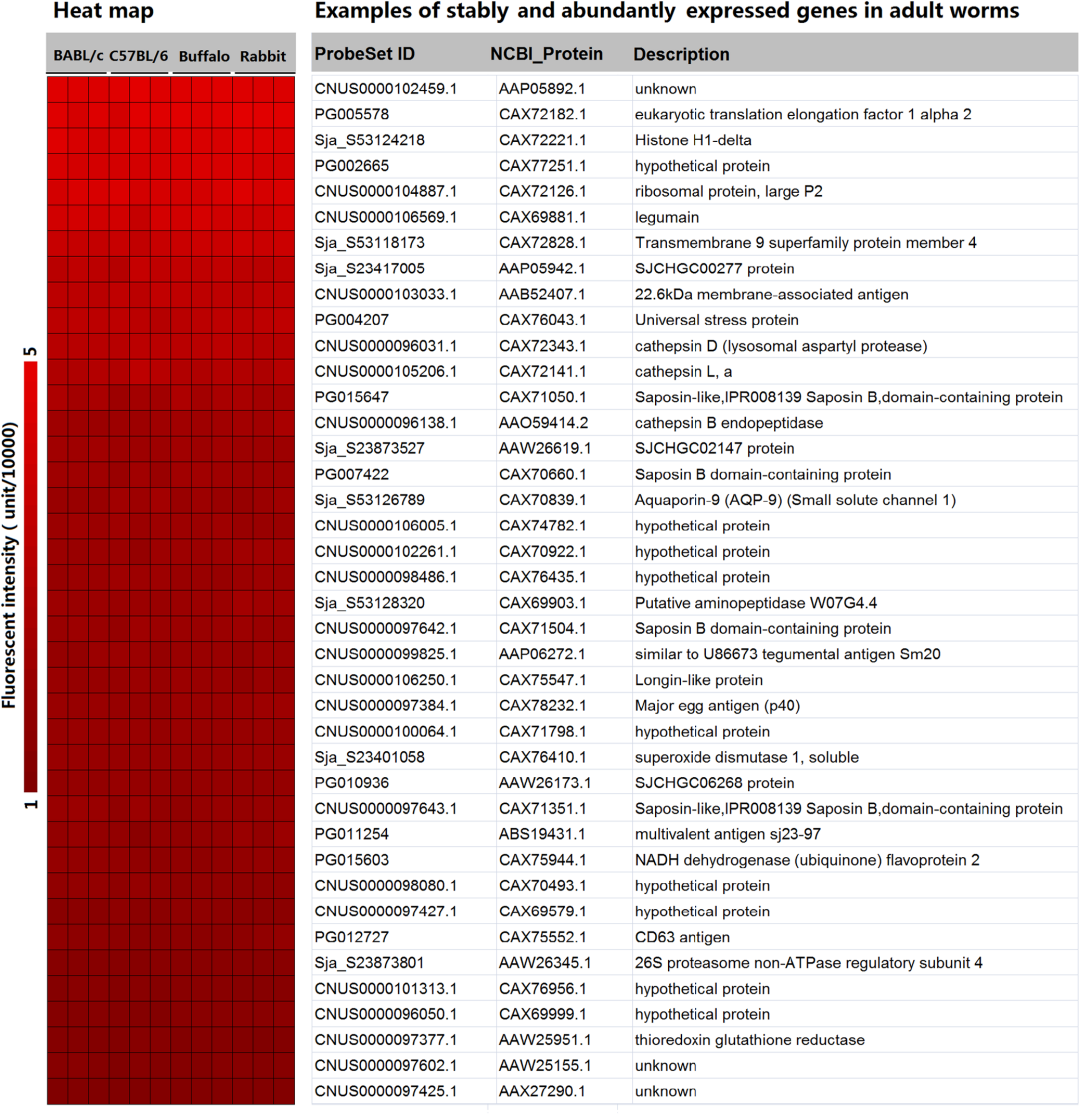
Examples of abundantly and stably expressed genes in adult worms from BALB/c mice, C57BL/6 mice, rabbits and water buffaloes. The heat map shows the normalized fluorescent intensity values of these genes abstracted from the microarray data. Three biological replicates were performed for each worm sample.

Using microarray fluorescent intensity ≥10,000 as the cut-off value, 1,540 gene products that exhibited the lowest coefficient of variation (0.1) in expression among the worms isolated from different hosts were obtained (S5 Table). A list of selected genes with various functions is presented in Fig 7. The gene annotation results revealed that many of these stably and abundantly expressed genes are conventional housekeeping genes, some of which have been proved to be constitutively expressed across the schistosome lifecycle, such as eukaryotic translation factors, ribosomal proteins, histone proteins, tubulins, proteasome subunits, and NADH dehydrogenase subunits [19, 50]. The genes encoding *S*. *japonicum* 26S proteasome non-ATPase regulatory subunit 4 (PSMD4) and NADH dehydrogenase (ubiquinone) flavoprotein 2 (NDUFV2) have been extensively used as references for real-time PCR analysis [19,20,51,52]. Comparative genomics analysis indicated that a substantial proportion of these schistosome genes share various sequence identity with their homologous counterparts in *H*. *sapiens*. Importantly, crystal structures of some of the human proteins are available, providing a foundation for the future screening of compounds that specifically target schistosome proteins based on structural disparity (S5 Table). Some of the genes identified in the present study have been previously characterized as important functional genes for schistosome and potential anti-schistosome targets, although some of these genes were developmentally regulated during the lifecycle. For example, some genes encoding schistosome proteases (legumain, cathepsin B, C, D and L) that were up-regulated from cercariae to adult worms play key roles in obtaining nutrients from the host [20,53]. The schistosome tegumental aquaporin gene, which is important for parasite osmotic regulation, is most highly expressed during the intravascular life stages [54], and the schistosome thioredoxin glutathione reductase gene has been validated as a potential drug target for schistosomiasis chemotherapy [55,56].

The schistosome tegument is a dynamic host-interactive surface that is involved in nutrition, immune evasion/modulation, excretion, osmoregulation, sensory reception, and signal transduction [54,57]. The most promising schistosome vaccine candidates are proteins located on the surface of the worms, such as the tegument proteins TSP-2 (tetraspanin protein) and Sm29 [58–60]. In *S*. *mansoni*, TSP-2, which plays a prominent part in the parasite tegument development and maturation, seems to be an effective vaccine antigen against the blood fluke [61,62]. A set of 107 genes with putative signal peptides and 276 genes with putative transmembrane helices were identified by sequence interrogation with SignalP 4.1 and TMHMM 2.0. These proteins may be secreted or surface-exposed and thus capable of interacting with the external environment. More importantly, we determined that a significant number of these stably expressed genes in adults were peculiar to *Schistosoma* species or the phylum Platyhelminthes and have no identity to genes in other organisms. Encouragingly, one of these *Schistosoma-* or phylum Platyhelminthes-specific genes (SjSP-13) has been identified as a novel biomarker for immunodiagnosis of *S*. *japonicum* infection exhibiting extremely high sensitivity (90.4%) and specificity (98.9%), with nearly no cross-reactivity with other fluke infections [63]. Therefore, *Schistosoma-* or phylum Platyhelminthes-specific genes, particularly secreted and transmembrane proteins, may be regarded as novel potential anti-parasite drug targets or vaccine candidates for future studies.

### Phylogenetic analysis and global expression profiling of the schistosome venom-allergen-like gene family

**Fig 8:**
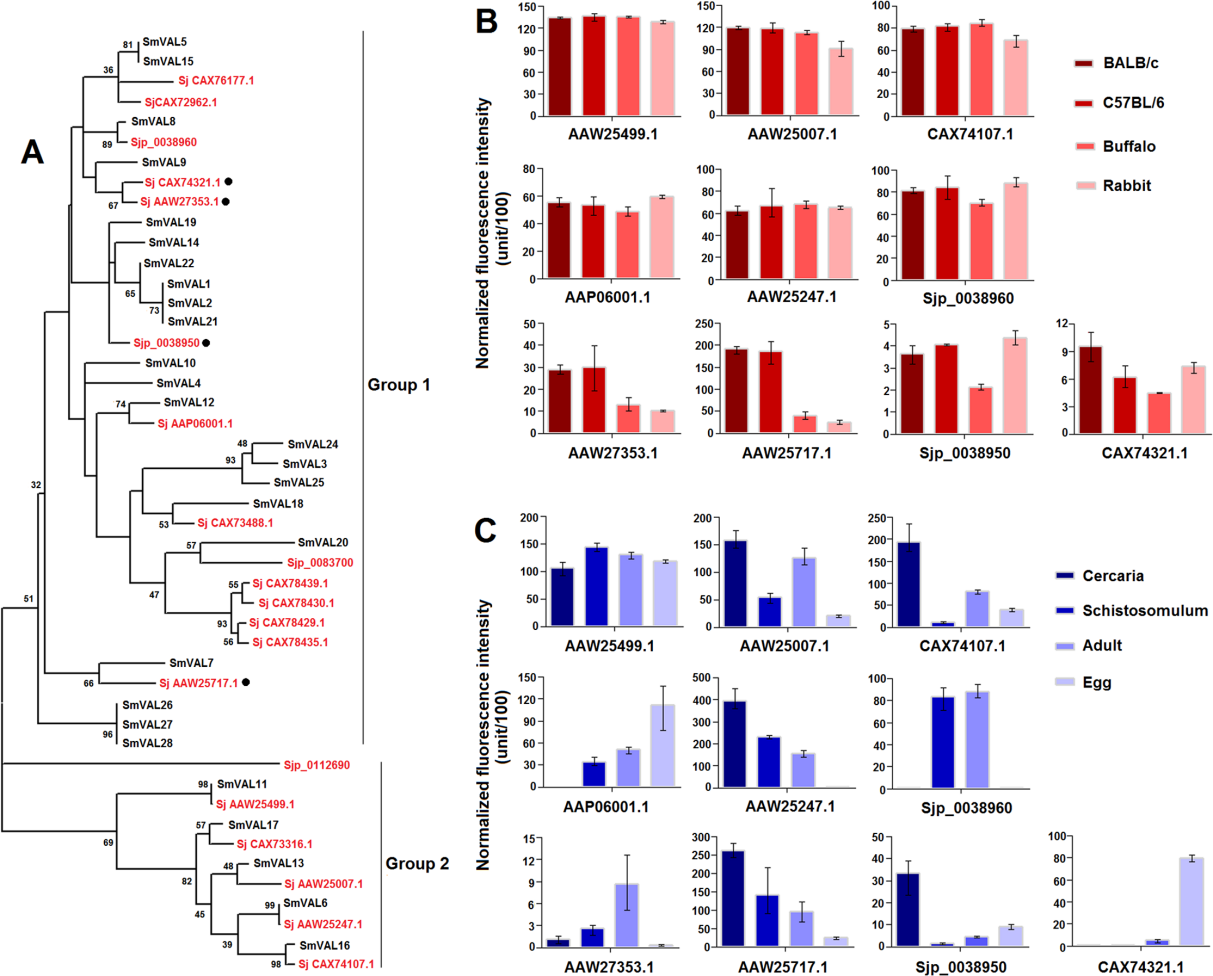
Molecular phylogenetic relationships and expression profiles of *Schistosoma* VAL genes. **(A)** The unrooted phylogenetic tree was generated using MEGA5.0 and the neighbor-joining method with 1000 bootstrap replicates. The tree was divided into two phylogenetic groups, and the bootstrap values are shown at the nodes. *S*. *japonicum* genes are indicated in red letters, and *S*. *mansoni* genes are indicated in black letters. ● represents differentially expressed genes in adult worms from the different mammalian hosts. **(B)** Transcription profiles of ten SjVALs in adult worms from BALB/c mice, C57BL/6 mice, rabbits and water buffaloes. **(C)** Transcription profiles of ten SjVALs in egg, schistosomulum, adult worm (from rabbit) and cercaria. The average gene expression values of three biological replicates were obtained from the microarray data. The upper and lower error bars represent the maximum and minimum values of the three biological replicates.

Structurally related members of the sperm-coating protein/Tpx-1/Ag5/PR-1/Sc7 (SCP/TAPS; Pfam: PF00188) family have been characterized in a wide range of eukaryotes, including parasites [64]. Parasitic helminth SCP/TAPS proteins have been proposed to play important biological roles in the transition from the free-living to the parasitic stage during the invasion of the mammalian host [64,65]. In *S*. *mansoni*, this family is termed venom-allergen-like proteins (SmVALs) and comprises at least 28 members [32]. SmVALs can be divided into two groups and exhibit various gene expression patterns throughout the entire life-cycle, including genes exclusively expressed in stages involved in intermediate host invasion or definitive host invasion and ubiquitously expressed genes [32]. Because of their potential functional classification, expression patterns and localization, SmVALs have been proposed as potential drug targets and vaccine candidates, and some members have been well characterized recently [66–68]. For instance, Farias et al. have demonstrated that SmVAL4 and SmVAL26 stimulate differential allergic responses in a murine model of airway inflammation [66]; and in a recent study, egg-derived SmVAL9 was found to carry an N-linked glycan containing a schistosome-specific difucosyl element and function as an immunogenic target during chronic murine schistosomiasis [68]. In our study, 20 non-redundant *S*. *japonicum* venom-allergen-like protein (SjVAL) genes were identified in the genome. In order to illuminate the phylogenetic relationships of VALs in the two *Schistosoma* species, we constructed a phylogenetic tree using all the full-length protein sequences, except SmVAL23 of which conserved domain was incomplete. The result revealed that all the *Schistosoma* VALs could also be divided into two groups: sequences in group one contain the SCP_euk conserved domain (cd05380) and only a partial sequence had homologous counterparts between the two *Schistosoma* species; sequences in group two contain the SCP_GAPR-1_like conserved domain(s) (cd05382) and the majority had homologous counterparts (Fig 8A). Microarray analysis revealed that ten SjVAL genes were transcribed in adult worms, including four genes (AAW25717.1, AAW27353.1, CAX74321.1 and Sjp_0038950) that were differentially transcribed in worms from the four hosts and six genes (AAP06001.1, Sjp_0038960, AAW25247.1, AAW25499.1, AAW25007.1 and CAX74107.1) that were stably expressed in worms from the four hosts (Fig 8B). All four differentially expressed SjVAL genes belonged to group one, and three encoded proteins with a putative signal peptide, which may be excreted/secreted proteins and interact with their immediate environment. Moreover, further expression analysis, based on our recently released *S*. *japonicum* microarray data for cercaria, schistosomulum, adult worm and egg (GEO accession number: GSE57143) [20] indicated that these ten genes exhibited diverse expression patterns in the four developmental stages (Fig 8C). It suggests that the majority of *Schistosoma* VALs, if not all, may be susceptible to the environment in the life cycle implying a role in function of host adaptation. In *S*. *mansoni*, six SmVAL genes were demonstrated to be highly expressed in the cercaria by microarray analysis of larval stages associated with infection of the mammalian host, implying that the functions of these enigmatic genes are mostly associated with entry into the mammalian host [69]. Specifically, the transcription of gene Sjp_0038960 is restricted to the schistosomulum and adult stages, suggesting that this gene is important for schistosome parasitism in the mammalian host. Notably, only one gene, AAW25499.1, was constantly expressed among the four developmental stages, indicating that this gene may play a fundamental role in the schistosome life cycle. Indeed, AAW25499.1 was also stably expresses in worms from different mammalian hosts.

In conclusion, we systematically compared the gene expression profiles of schistosomes from laboratory animal hosts (BALB/c mice, C57BL/6 mice and rabbits) and the natural host (water buffaloes). The global transcriptional profiles of schistosomes from the four different hosts were generally coincident with each other. Meanwhile, a panel of differentially expressed genes was identified, which mainly involved in signal transduction and metabolism processes. A set of *Schistosoma-* or phylum Platyhelminthes-specific genes were differentially or stably expressed in schistosomes from the four different hosts and should be targeted in future hypothesis-driven functional studies. The findings of this study provide a rational basis for schistosomiasis research in different laboratory animals and natural mammalian host at the transcriptional level and a valuable resource for the screening of anti-schistosomal intervention targets.

## Supporting Information

S1 Table List of primers used for real-time PCR analysis. The primers for reference gene PSMD4 were obtained from Liu *et al*. [19].
(XLSX)

S2 Table The statistical analysis of differentially expressed genes identified by pairwise comparisons of adult worms from BALB/c mice, C57BL/6 mice, rabbits and buffaloes. (XLSX)

S3 Table Details of the differentially expressed gene products in adult worms from BALB/c mice, C57BL/6 mice, rabbits and buffaloes. (XLSX)

S4 Table The expression profiles extracted from our microarray database for genes which were differentially expressed in schistosomes from water buffaloes, yellow cattle and goats [7,8]. (XLSX)

S5 Table Details of the abundantly and stably expressed gene products in adult worms from BALB/c mice, C57BL/6 mice, rabbits and buffaloes. (XLSX)

S1 Fig Gene expression patterns of *S*. *japonicum* TFPI gene (CAX69506.1) in worms from different hosts and developmental stages. **(A)** Gene expression pattern of *S*. *japonicum* TFPI gene in adult worms from BALB/c mice, C57BL/6 mice, water buffaloes and rabbits. **(B)** Gene expression pattern of *S*. *japonicum* TFPI gene in egg, schistosomulum, adult worm (from rabbits) and cercaria. The average gene expression values of three biological replicates were obtained from the microarray data. The upper and lower error bars represent the maximum and minimum values of the three biological replicates.
(TIF)
