# Supplementary figures and images for: Comparative Analysis of Transcriptional Profiles of Adult Schistosoma japonicum from Different Laboratory Animals and the Natural Host, Water Buffalo
Source: PLoS Negl Trop Dis. 2015 Aug 18;9(8):e0003993. doi: 10.1371/journal.pntd.0003993 (PMC4540470; doi:10.1371/journal.pntd.0003993)

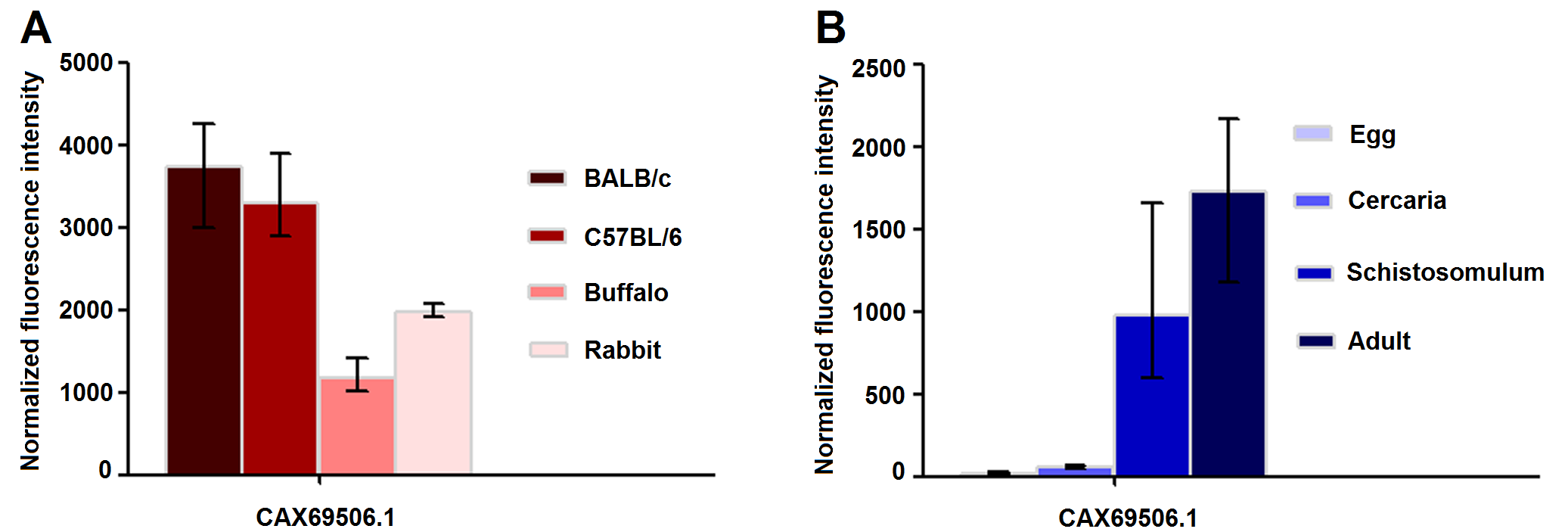

Supplement: S1 Fig — (A) Gene expression pattern of S. japonicum TFPI gene in adult worms from BALB/c mice, C57BL/6 mice, water buffaloes and rabbits. (B) Gene expression pattern of S. japonicum TFPI gene in egg, schistosomulum, adult worm (from rabbits) and cercaria. The average gene expression values of three biological replicates were obtained from the microarray data. The upper and lower error bars represent the maximum and minimum values of the three biological replicates. (TIF) [file pntd.0003993.s006.tif]
